# High MYC mRNA Expression Is More Clinically Relevant than MYC DNA Amplification in Triple-Negative Breast Cancer

**DOI:** 10.3390/ijms21010217

**Published:** 2019-12-28

**Authors:** Eriko Katsuta, Li Yan, Takashi Takeshita, Kerry-Ann McDonald, Subhamoy Dasgupta, Mateusz Opyrchal, Kazuaki Takabe

**Affiliations:** 1 Breast Surgery, Department of Surgical Oncology, Roswell Park Comprehensive Cancer Center, Buffalo, NY 14263, USA; eriko.katsuta@roswellpark.org (E.K.); takashi.takeshita@roswellpark.org (T.T.);; 2 Department of Biostatistics and Bioinformatics, Roswell Park Comprehensive Cancer Center, Buffalo, NY 14263, USA; li.yan@roswellpark.org; 3 Department Cell Stress Biology, Roswell Park Comprehensive Cancer Center, Buffalo, NY 14263, USA; subhamoy.dasgupta@roswellpark.org; 4 Department of Medicine, Roswell Park Comprehensive Cancer Center, Buffalo, NY 14263, USA; m.opyrchal@wustl.edu; 5 Division of Oncology, Department of Internal Medicine, Washington University in St. Louis, St. Louis, MO 63110, USA; 6 Department of Surgery, University at Buffalo Jacobs School of Medicine and Biomedical Sciences, The State University of New York, Buffalo, NY 14203, USA; 7 Department of Breast Surgery and Oncology, Tokyo Medical University, Tokyo 1608402, Japan; 8 Department of Surgery, Yokohama City University, Yokohama 2360004, Japan; 9 Department of Surgery, Niigata University Graduate School of Medical and Dental Sciences, Niigata 9518510, Japan; 10 Department of Breast Surgery, Fukushima Medical University School of Medicine, Fukushima 9601295, Japan

**Keywords:** MYC, amplification, expression

## Abstract

DNA abnormalities are used in inclusion criteria of clinical trials for treatments with specific targeted molecules. MYC is one of the most powerful oncogenes and is known to be associated with triple-negative breast cancer (TNBC). Its DNA amplification is often part of the targeted DNA-sequencing panels under the assumption of reflecting upregulated signaling. However, it remains unclear if MYC DNA amplification is a surrogate of its upregulated signaling. Thus, we investigated the difference between MYC DNA amplification and mRNA high expression in TNBCs utilizing publicly available cohorts. MYC DNA amplified tumors were found to have various mRNA expression levels, suggesting that MYC DNA amplification does not always result in elevated MYC mRNA expression. Compared to other subtypes, both MYC DNA amplification and mRNA high expression were more frequent in the TNBCs. MYC mRNA high expression, but not DNA amplification, was significantly associated with worse overall survival in the TNBCs. The TNBCs with MYC mRNA high expression enriched MYC target genes, cell cycle related genes, and WNT/β-catenin gene sets, whereas none of them were enriched in MYC DNA amplified TNBCs. In conclusion, MYC mRNA high expression, but not DNA amplification, reflects not only its upregulated signaling pathway, but also clinical significance in TNBCs.

## 1. Introduction

Clinical targeted DNA-sequencing to detect genomic alterations, including mutation and copy number alterations, has become routine in clinical practice for multiple cancers. Increasingly, clinical trials are using abnormalities in DNA as criteria for enrolling patients in order to test novel targeted therapy options and choose the next line of therapy. However, the interpretation of the results in each gene remains understudied. 

MYC is one of the most powerful oncogenes encoding transcription factor. MYC forms a heterodimer with MYC-associated factor X and binds to the E box of its target genes to regulate gene expressions [1]. MYC regulates the expression of many genes involved in cellular proliferation, transformation, angiogenesis, and cell cycle control, such as the E2F family and the G2/M check point genes [1,2,3]. MYC promotes oncogenesis by regulating cell growth and metabolism [4]. MYC is also associated with stem cell features such as chemo-resistance and self-renewal capability [5]. Thus, MYC transcription is strictly regulated by many signaling pathways including WNT/β -catenin, Notch, and TGF-β pathways [1]. MYC is one of the most dysregulated oncogenes, including translocation and copy number alteration, in various types of cancers [4,6,7,8,9,10]. Inactivation of MYC has been demonstrated to lead to tumor regression in murine models [11,12,13]. MYC DNA amplification is part of the eligibility criteria of some clinical trials because drugs that target the MYC pathway, such as ibrutinib and prexasertib, have been developed.

It has been reported that the proportion of MYC DNA amplification as well as elevated mRNA expression is higher in triple-negative breast cancers (TNBCs) compared to other breast cancer subtypes [7,14,15,16]. However, the clinical relevance of MYC DNA amplification and mRNA high expression in TNBC has not been fully elucidated. MYC DNA amplification is often a part of clinical targeted DNA-sequencing panels under the assumption that it reflects higher mRNA expression, and thus a higher signaling of the MYC pathway. However, whether MYC DNA amplification consistently reflects higher mRNA expression or the downstream signaling pathway in TNBCs remains unknown.

In this study, we aimed to investigate the clinical significance of MYC DNA amplification and mRNA high expression in TNBCs through the use of two large publicly available cohorts, The Cancer Genome Atlas (TCGA) [17] and METABRIC [18].

## 2. Results

### 2.1. MYC DNA Amplified Tumors Do Not Always Express High Levels of MYC mRNA

**Figure 1:**
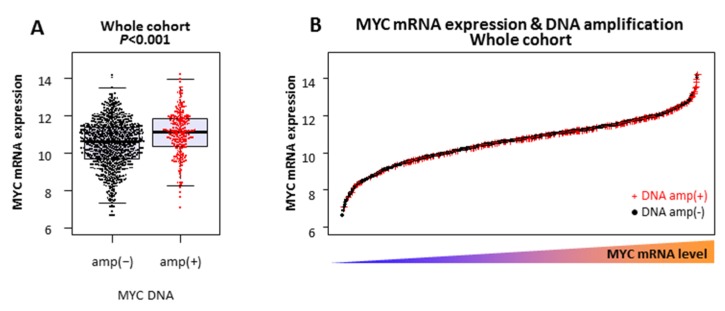
MYC mRNA expression and DNA amplification in The Cancer Genome Atlas (TCGA) whole breast cancer cohort: (**A**) MYC mRNA expression levels of MYC DNA non-amplified (amp(−)) and amplified (amp(+)) tumors, and (**B**) MYC mRNA expression levels of MYC DNA non-amplified (DNA amp(−) in black) and amplified (DNA amp(+) in red) tumors.

There were 1075 breast cancer tumors with both mRNA expression and DNA copy number alteration data in TCGA. Among them, 228 (21.2%) were MYC DNA amplified and the others (78.8%) were MYC DNA non-amplified tumors. As expected, the MYC mRNA expression level was statistically higher in the MYC DNA amplified tumors compared to the MYC DNA non-amplified tumors (*p* < 0.001) (Figure 1A). However, the mRNA expression levels were largely overlapped between DNA amplified and non-amplified tumors (Figure 1A,B). Another independent METABRIC cohort was analyzed and it validated that not all MYC DNA amplified tumors have elevated MYC mRNA expression (Figure S1A,B). Further, seven of representative human TNBC cell lines were also analyzed (Figure S1C). HCC1143 and MDA-MB-436 were found to have MYC DNA amplification. Indeed, HCC1143 showed the highest MYC mRNA level; however, MDA-MB-436 MYC mRNA expression was the third from the bottom among seven cell lines. This result suggests that MYC DNA amplification does not always result in elevated MYC mRNA expression.

### 2.2. Neither MYC DNA Amplification nor MYC mRNA High Expression Is Associated With Survival in the Breast Cancer Whole Cohort

**Figure 2:**
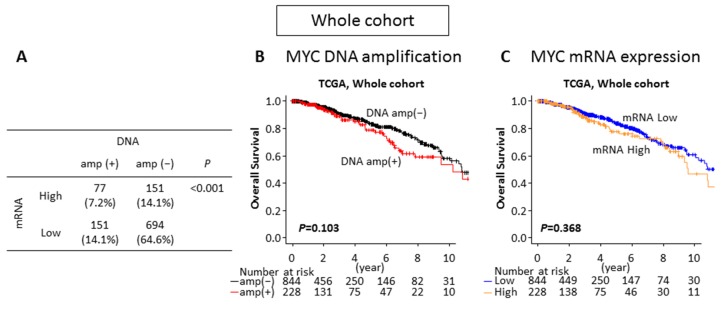
The impacts of MYC DNA amplification and mRNA expression on patient survival in TCGA whole breast cancer cohort. (**A**) Patients proportion of each group by MYC DNA amplification and mRNA expression. (**B**) Overall survival comparing the MYC DNA non-amplified (DNA amp(−) in black) and amplified (DNA amp(+) in red) tumors. (**C**) Overall survival comparing the MYC mRNA low (mRNA low in blue) and high (mRNA high in orange) expressing tumors.

In order to investigate the impact of MYC DNA amplification and mRNA expression on patient survival in the whole TCGA cohort, the patients were divided into two groups. An MYC mRNA high and an MYC mRNA low expression group were created, which were distributed as the same proportion of DNA amplified (21.2%) and non-amplified tumors (78.8%), respectively. Out of 1075 patients, 3 patients did not have overall survival (OS) data and were excluded from the survival analyses. Among 1072 patients, the distribution of MYC DNA amplified and mRNA high expressing, DNA amplified and mRNA low expressing, DNA non-amplified and mRNA high expressing, and DNA non-amplified and mRNA low expressing tumors were 77 (7.2%), 151 (14.1%), 151 (14.1%), and 694 (64.6%), respectively (Figure 2A). Although one third of MYC DNA amplified tumors expressed high levels of MYC mRNA (*p* < 0.001), the majority (66.2%) of MYC DNA amplified tumors did not (Figure 2A). Interestingly, there was no statistically significant survival difference between the MYC DNA non-amplified and the amplified tumors (*p* = 0.103) (Figure 2B), as well as between the MYC mRNA low and high expressing tumors (*p* = 0.368) in the whole cohort (Figure 2C).

### 2.3. Distributions of MYC DNA Amplified and mRNA High Expressing Tumors Are Different in Each Subtype

**Figure 3:**
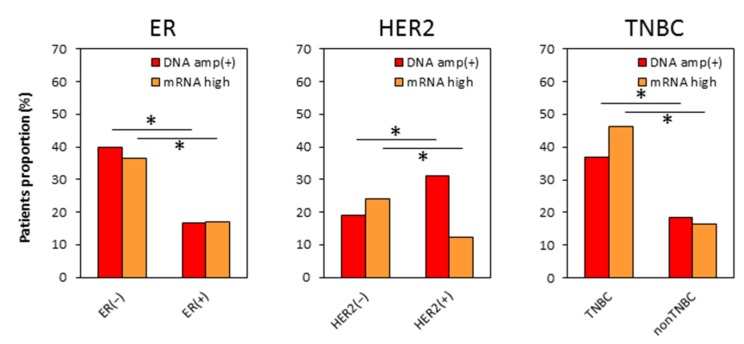
The patient distribution of the MYC DNA amplification and mRNA high expression by each subtype in TCGA breast cancer patients. MYC DNA amplified tumors (DNA amp(+) as red column) and MYC mRNA high expression tumors (mRNA high as orange column).

To determine if the clinical impact of MYC DNA amplification or mRNA expression differs by breast cancer subtype, we analyzed the distribution of MYC DNA amplified and mRNA high expressing tumors in each breast cancer subtype. There was a higher proportion of MYC DNA amplified tumors (*p* < 0.001) as well as MYC mRNA high expressing tumors (*p* < 0.001) in estrogen receptor (ER) negative tumors (*p* < 0.001) and TNBCs (*p* < 0.001) (Figure 3). These results were consistent with previous reports that MYC DNA amplification is more frequent and mRNA expression level is higher in TNBC [7,8,19]. However, there was a higher proportion of MYC DNA amplified tumors in human epidermal growth factor receptor-2 (HER2) positive tumors (*p* < 0.001), whereas MYC mRNA high expressing tumors were higher in HER2 negative tumors (*p* < 0.001) (Figure 3). These findings suggest that both MYC DNA amplification and mRNA expression highly associate with ER negative tumors, but they differ in relationship to HER2 overexpression.

### 2.4. No Difference in Patient Demographics by MYC DNA Amplification or by MYC mRNA Expression in TNBC

**Figure 4:**
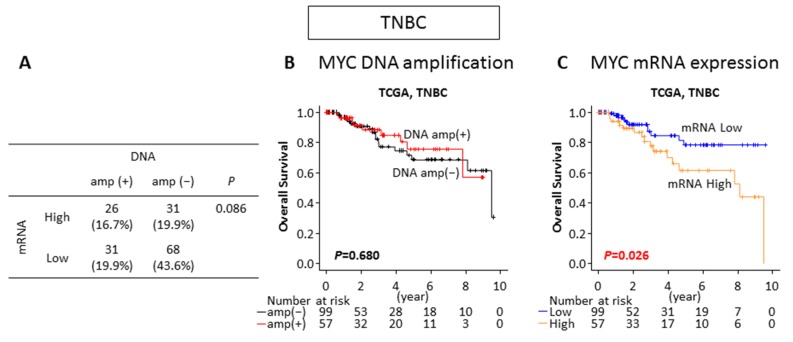
The impacts of the MYC DNA amplification and mRNA expression on patient survival in TCGA TNBC cohort. (**A**) The proportion of each group by MYC DNA amplification and mRNA expression in TNBC. (**B**) Overall survival comparing the MYC DNA non-amplified (DNA amp(−) in black) and amplified (DNA amp(+) in red) tumors in TNBC. (**C**) Overall survival comparing the MYC mRNA low (mRNA low in blue) and high (mRNA high in orange) expressing tumors in TNBC.

We investigated whether TNBC patients with MYC DNA amplification or mRNA high expression have differences in their clinical characteristics. Out of 156 TNBC patients in TCGA breast cancer cohort, 57 (36.5%) tumors were found to be MYC DNA amplified and the same proportion of patients (36.5%) were classified as MYC mRNA high expressing tumors based on expression level. As shown in Figure 4A, the distribution of the MYC DNA amplified and mRNA high expressing, DNA amplified and mRNA low expressing, DNA non-amplified and mRNA high expressing, and DNA non-amplified and mRNA low expressing tumors were 26 (16.7%), 31(19.9%), 31 (19.9%), and 68 (43.6%), respectively. Interestingly, the proportion of MYC mRNA high expression tumors was not statistically different between the MYC DNA amplified and the non-amplified tumors (*p* = 0.086) (Figure 4A). This result suggests that MYC DNA amplification is not directly associated with a high MYC mRNA level, similar to the whole cohort data. To investigate whether MYC DNA amplification or the mRNA expression level correlates with the protein level, we analyzed Reverse Phase Protein Array (RPPA) data of TNBCs in TCGA for MYC protein levels and correlated it with the MYC DNA amplification status and mRNA expression. MYC protein levels were not significantly different between non-amplified and amplified TNBCs (*p* = 0.409), as well as with the mRNA low and high TNBCs (*p* = 0.138) (Figure S2). These results are consistent with previous reports that protein levels of transcription factors are intricately regulated by post-translational modifications to maintain protein stability and activity, and hence, often do not correlate with mRNA levels [20]. We then compared clinical demographics between the MYC DNA amplified and non-amplified TNBCs, as well as the MYC mRNA high and low expressing TNBCs. It was initially assumed that factors that influence cancer aggressiveness, such as TNM stage and race, associates with MYC status. However, MYC DNA amplification or MYC mRNA high expression did not associate with any of analyzed clinical characteristics in TNBCs (Table 1).

### 2.5. MYC mRNA High Expression but Not DNA Amplification Is Associated With Worse Survival in TNBC

We then analyzed the overall survival (OS) of the TNBC patients by the MYC DNA amplification status and the mRNA expression level. Interestingly, there was no significant difference in OS between the MYC DNA non-amplified and amplified tumors (*p* = 0.680) (Figure 4B). On the other hand, the MYC mRNA high expressing tumors showed significantly worse OS compared to the low expressing tumors in TNBCs (*p* = 0.026) (Figure 4C). These findings suggest that prognosis is associated with the mRNA expression level of MYC, but not the DNA amplification in TNBC.

### 2.6. MYC Targets, Cell Cycle Related, and WNT/ β-Catenin Signaling Gene Sets Are Enriched in MYC mRNA High Expression, but Not DNA Amplification, in TNBCs

**Figure 5:**
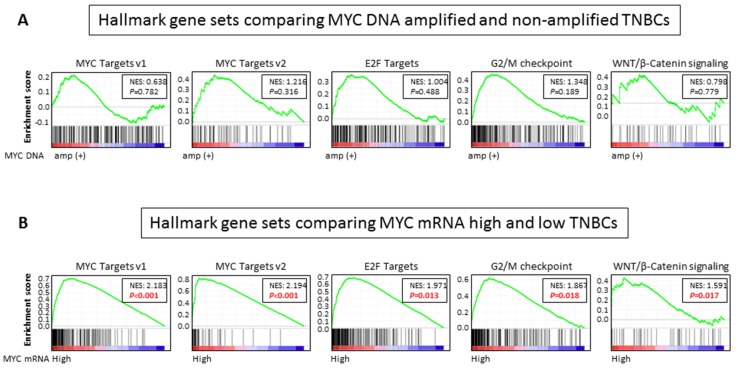
A Gene Set Enrichment Analysis (GSEA) comparing (**A**) MYC DNA amplified and non-amplified TNBCs, and (**B**) MYC mRNA high and low expressing TNBCs in TCGA.

In order to explore underlying mechanisms of association between MYC mRNA high expression and poor prognosis in TNBC, Gene Set Enrichment Analysis (GSEA) was conducted utilizing 50 hallmark gene sets [21] (Figure 5). We found that MYC targets gene sets (v1: Normalized Enrichment Score (NES) = 2.183, *p* < 0.001; v2: NES = 2.194, *p* < 0.001) [21] as well as cell cycle related gene sets that are known to be regulated by MYC [22,23] (E2F targets: NES = 1.971, *p* = 0.013; G2/M check point: NES = 1.867, *p* = 0.018) were enriched in the MYC mRNA high expressing TNBCs (Figure 5B). In addition, the WNT/β-catenin gene set, which regulates the MYC signaling pathway, was also enriched in the MYC mRNA high expressing TNBCs (NES = 1.591, *p* = 0.017) (Figure 5B). These findings were validated in METABRIC cohort (Figure S3A), in which all of the gene sets that were enriched in TCGA were also enriched in MYC mRNA high expressing TNBCs. Further, MYC high expressing cell lines showed higher expressions of cell cycle related genes, including Cyclin dependent kinase 4(CDK4) and cyclin D1 (CCND1) (Figure S3B), as well as WNT/ β-Catenin signaling genes, including FZD1 and FZD8, regardless of MYC DNA amplification status (Figure S3C). On the other hand, no gene set was enriched among 50 hallmark gene sets in the MYC DNA amplified tumors comparing non-amplified tumors in TNBC (Figure 5A). Together with the survival analyses results, these findings suggest that high expression of MYC mRNA, but not DNA amplification, reflects increased the MYC signaling, which is associated with a detrimental effect on clinical outcomes in TNBCs.

## 3. Discussion

In the current study, we found that MYC DNA amplification was not equivalent to high expression of MYC mRNA in either the whole breast cancer or TNBC cohort. In the whole cohort, neither MYC DNA amplification nor mRNA high expression was associated with patient survival. On the other hand, there were higher proportions of MYC DNA amplified and mRNA high expressing tumors in the TNBCs. Although there was no significant difference in any of the clinical demographics examined, mRNA high expression of MYC significantly associated with worse OS in the TNBCs with upregulated MYC targets, cell cycles, and WNT/β-catenin pathway related genes. MYC DNA amplification did not associate with any of the hallmark gene sets.

MYC, one of the most studied and powerful oncogenes, functions as a transcription factor [9], and approximately 15% of human genes are regulated by MYC [1]. In our results, MYC mRNA high expression was associated with upregulation of its target genes, whereas DNA amplification was not in TNBCs. Cell cycle related genes were also upregulated in the MYC mRNA high expressing TNBCs. It is well known that activation of the MYC signaling pathway leads to an increase in cell cycle activation [24]. MYC controls the G2/S transition by activating down-stream targets, such as CCNE and Cyclin dependent kinase (CDK) 2, and it dampens the effect of the CDK inhibitor [2]. MYC also promotes cell cycle progression by activation of the CDK4, CCND1, CDC25A, and E2F family [25]. Therefore, our results suggest that MYC mRNA high expression, not MYC DNA amplification, reflects the function of the MYC signaling pathway in TNBC.

MYC transcription itself is also regulated by many signaling pathways, including WNT/β-catenin signaling, which is one of the most frequent abnormalities in human cancers [8] and is associated with poor prognosis in breast cancer [26,27,28]. We found that the MYC mRNA high expressing TNBCs significantly enriched genes were involved in the WNT/β-catenin pathway, which evoked us to speculate that MYC mRNA high expression forms an amplification loop with the WNT/β-catenin pathway, whereas MYC DNA amplification may not. The impact of MYC DNA amplification on the prognosis of breast cancer is less consistent; some studies reported that MYC DNA amplification is associated with a worse prognosis [29,30], and the others reported no association [31]. Similarly, the association of MYC mRNA high expression and prognosis is also not consistent [32,33,34]. In our analyses of the whole cohort, MYC DNA amplification, as well as mRNA high expression, did not associate with prognosis. It is well known that there is a higher proportion of MYC DNA amplification and mRNA high expression in TNBC compared to other subtypes [1,8]; however, the impact of MYC DNA amplification and mRNA high expression in TNBC is not fully understood. In our results, we found that MYC mRNA high expression, but not DNA amplification, in TNBCs was associated with worse OS, which is consistent with previous reports showing that high expression of MYC mRNA is associated with worse prognosis in TNBCs [5]. These findings further imply that MYC mRNA expression, but not DNA amplification, represents true activation of the oncogene. This observation was supported by our data showing that the MYC DNA amplification did not associate with downstream MYC target genes, but with the MYC mRNA high expression that is significantly associated with MYC targets and cell cycle gene sets, which are known to be regulated by MYC target genes in TNBC.

Some of the studies suggested that the elevation of MYC mRNA expression is determined not only by its DNA amplification, but also other factors such as transcriptional regulation of mRNA. However, the biological difference between MYC DNA that is amplified and mRNA overexpressing tumors has not been fully demonstrated [1,8]. To our knowledge, this is the first report showing the difference between MYC DNA amplification and mRNA high expression in TNBCs.

Targeted sequencing of tumor tissue is becoming a more common component of oncology practices. There are multiple compounds in development that target MYC pathways. Currently, there are three actively enrolling clinical trials that have MYC DNA amplification in its eligibility criteria (clinicaltrials.gov). However, our results showed that MYC DNA amplification does not reflect upregulation of MYC signaling or outcome of TNBC patients. Thus, mRNA expression may be a better indicator of the MYC signaling upregulation, and thus, a predictive biomarker for response to targeted therapies designed to block MYC signaling. Indeed, it has been reported that high MYC mRNA in TNBCs is a potential indicator of a PIM1 kinase inhibitor in the preclinical model [35].

There are limitations to our study. First, this study was conducted using only a few publicly available cohorts due to availability of both DNA and mRNA sequencing data. Secondly, this study is based only on DNA amplification and mRNA expression of the primary tumor. In order to determine the role of MYC and to elucidate its molecular mechanism in TNBC, further experimental approach in both primary and metastatic tumors is needed.

In conclusion, we found that MYC DNA amplification does not always reflect high expression of MYC mRNA. Elevated MYC mRNA expression is associated with worse prognosis in TNBC with upregulated MYC target genes as well as cell cycle and WNT/β-catenin gene sets, whereas MYC DNA amplification is not. MYC mRNA expression levels are more reflective of the MYC signaling. Further studies should be focused on MYC mRNA expression in TNBC.

## 4. Materials and Methods

### 4.1. Data Acquisition and Pre-Processing

There are 1098 breast cancer patients in TCGA, which is a project supervised by the National Cancer Institute (NCI) and the National Human Genome Research Institute [17]. There are 1904 breast cancer patients in METABRIC, which is a cohort from Europe and Canada [18]. The gene expression level quantification data (mRNA expression z-score from RNA-sequence), copy-number alteration data from GISTIC, protein expression data from RPPA for TCGA cohort, gene expression level quantification data (mRNA expression z-score from microarray), and copy-number alteration data from GISTIC were downloaded through cBioportal (TCGA provisional and METABRIC datasets) [36,37]. Out of 1098, 1075 patients have both mRNA expression from the RNA sequence and DNA copy-number alteration data, and 1072 patients have OS data in the breast cancer cohort of TCGA. Patients were divided into either MYC DNA amplified tumors (GISTIC: 2) or MYC DNA non-amplified tumors (GISTIC: from −2 to 1) according to the definition of the amplification in the cBioportal, and then were divided into either MYC mRNA high or low expressing tumors based on the MYC mRNA level using a cutoff point to divide the same proportion of MYC DNA amplified and non-amplified tumors in the whole cohort as well as in the TNBC cohort. Since TCGA and METABRIC are de-identified publicly available cohorts, institutional review board approval was waived, which was the case with previous publications [38,39,40,41].

### 4.2. GSEA

GSEA was performed comparing the MYC DNA amplified and the non-amplified tumors, as well as the MYC mRNA high and the low expressing tumors among 50 hallmark gene sets [21] using software provided by the Broad Institute (http://software.broadinstitute.org/gsea/index.jsp), as we described previously [42,43,44,45,46].

### 4.3. Cell Culture and qPCR

Seven commonly used representative TNBC cell lines that have MYC DNA amplification status in the Cancer Cell Line Encyclopedia (CCLE) [47] and normal breast epithelial cell line MCF10A as a control were utilized in this study. MDA-MB-468, HCC38, HCC1143, BT549, and MCF10A were obtained from ATCC (Manassas, VA, USA). MDA-MB-231 was provided by Dr. John Ebos (Department of Cancer Genetics and Genomics, Roswell Park Comprehensive Cancer Center (RPCCC), Buffalo, NY, USA). BT20 and MDA-MB-436 were provided by Dr. Jianmin Zhang (Department of Cancer Genetics and Genomics, RPCCC). Cell culture conditions were shown in Table S1. Total RNA was extracted using the RNeasy Mini Kit (Qiagen, Velno, The Netherlands). Reverse transcription was carried out using High-Capacity cDNA Reverse Transcription Kit (Applied Biosystems, Waltham, MA, USA) according to the manufacturer’s instructions. GAPDH was used as an internal control. Sequences of primers were shown in Table S2. Data were analyzed using the ΔΔCt method and were normalized by the normal breast epithelial cell line, MCF10A. We utilized the top 2 MYC mRNA high expression cell lines, HCC1143 and MDA-MB-231, as MYC mRNA high expressing cell lines, and the bottom 3, BT20, BT549 and MDA-MB-436, as MYC mRNA low expressing cell lines.

### 4.4. Statistical Analysis

Gene expression differences were analyzed using the Student’s t-test, the survival differences were analyzed using Kaplan–Meier curves with the log-rank test, and the clinical demographics were compared using Fisher’s exact test. Two-sided *p* < 0.05 was considered as statistically significant for all tests. All statistical analyses were performed using R software (http:///www.r-project.org/) and Bioconductor (http://bioconductor.org/).

## Figures and Tables

**Table 1:** Clinical demographics by MYC DNA amplification and mRNA expression in TCGA Triple-Negative Breast Cancers (TNBCs).

	MYC DNA		*p*	MYC mRNA		*p*
	amp(+)	amp(−)		High	Low	
**Age**						
<60	36 (35.3%)	66 (64.7%)	0.728	40 (39.2%)	62 (60.8%)	0.385
>60	21 (38.9%)	33 (61.1%)		17 (31.5%)	37 (68.5%)	
**Race**						
White	33 (37.1%)	56 (62.9%)	0.833	26 (29.3%)	63 (70.8%)	0.167
Black	20 (37.0%)	34 (63.0%)		24 (44.4%)	30 (55.6%)	
Asian	2 (25.0%)	6 (75.0%)		3 (37.5%)	5 (62.5%)	
**Menopause Status**						
Pre	16 (44.4%)	20 (55.6%)	0.227	11 (30.6%)	25 (69.4%)	0.837
Post	32 (32.7%)	66 (67.3%)		33 (33.7%)	65 (66.3%)	
**pT**						
T1/2	50 (36.8%)	86 (63.2%)	>0.999	49 (36.0%)	87 (64.0%)	0.805
T3/4	7 (35.0%)	13 (65.0%)		8 (40.0%)	12 (60%)	
**pN**						
N0	36 (35.3%)	66 (64.7%)	0.728	33 (32.4%)	69 (67.6%)	0.163
N1/2/3	21 (38.9%)	33 (61.1%)		24 (44.4%)	30 (55.6%)	
**M**						
M0	49 (37.1%)	83 (62.9%)	>0.999	47 (35.6%)	85 (64.4%)	>0.999
M1	1 (50.0%)	1 (50.0%)		1 (50.0%)	1 (50.0%)	
**Stage**						
Stage I/II	48 (37.8%)	79 (62.2%)	0.827	46 (36.2%)	81 (63.8%)	0.827
Stage III/IV	9 (34.6%)	17 (65.4%)		10 (38.5%)	16 (61.5%)	
**Histology**						
IDC	51 (38.6%)	81 (61.4%)	0.409	47 (35.6%)	85 (64.4%)	>0.999
ILC	1 (16.7%)	5 (83.3%)		2 (33.3%)	4 (66.7%)	

IDC: Infiltrating Ductal Carcinoma, ILC: Infiltrating Lobular Carcinoma.

## Supplementary Materials

The following are available online at https://www.mdpi.com/1422-0067/21/1/217/s1.

## Abbreviations

TNBC	triple-negative breast cancer
TCGA	The Cancer Genome Atlas
OS	overall survival
ER	estrogen receptor
HER2	human epidermal growth factor receptor-2
GSEA	Gene Set Enrichment Analysis
NES	Normalized Enrichment Score
CDK	Cyclin dependent kinase
CCN	Cyclin
NCI	National Cancer Institute
