# Supplementary material for: High MYC mRNA Expression Is More Clinically Relevant than MYC DNA Amplification in Triple-Negative Breast Cancer
Source: Int J Mol Sci. 2019 Dec 28;21(1):217. doi: 10.3390/ijms21010217 (PMC6981812; doi:10.3390/ijms21010217)
Supplement: Supplementary file 1 [file ijms-21-00217-s001.pdf]

# Supplementary Materials:

# High MYC mRNA Expression Is More Clinically Relevant than MYC DNA Amplification in Triple-Negative Breast Cancer

**Eriko Katsuta [1], Li Yan [2], Takashi Takeshita [1], Kerry-Ann McDonald [1], Subhamoy Dasgupta [3], Mateusz Opyrchal [4,5] and Kazuaki Takabe [1,6,7,8,9,10,*]**

**Table S1.** List of culture medium.

| Cell Line | Medium |
|---|---|
| BT20 | MEM + 10% FBS |
| BT549 | RPMI-1640 + 10% FBS |
| HCC38 | RPMI-1640 + 10% FBS |
| HCC1143 | RPMI-1640 + 10% FBS |
| MDA-MB-231 | DMEM + 10% FBS |
| MDA-MB-436 | DMEM + 10% FBS + 10 μg/ml insulin + 16 μg/ml glutathione |
| MDA-MB-468 | DMEM + 10% FBS |
| MCF10A | DMEM/F12 + 5% Horse Serum + 20 ng/ml EGF + 0.5 mg/ml Hydrocortisone + 10 μg/ml Insuline |

MEM: Eagle's minimal essential medium, RPMI: Roswell Park Memorial Institute, DMEM: Dulbecco's Modified Eagle, FBS: Fetal Bovine Serum.

**Table S2.** qPCR primer sequence.

| Gene | F | R |
|---|---|---|
| MYC | TTTTTCGGGTAGTGGAAAACC | TTCCTGTTGGTGAAGCTAACG |
| CDK4 | TTTGTGGCCCTCAAGAGTGT | CCTCCAGTCGCCTCAGTAAA |
| CCND1 | GGTAGATGTGTAACCTCTTCACCTTA | GCACGCTACGCTACTGTAACC |
| FZD1 | GGTGCCCTCCTACCTCAACT | ATACACCTTGGTCGGCTCAC |
| FZD8 | TCGGTCATCAAGCAACAGG | GGTGTAGAGCACGGTGAACA |
| GAPDH | AAGGTGAAGGTCGGAGTCAAC | GGGGTCATTGATGGCAACAATA |

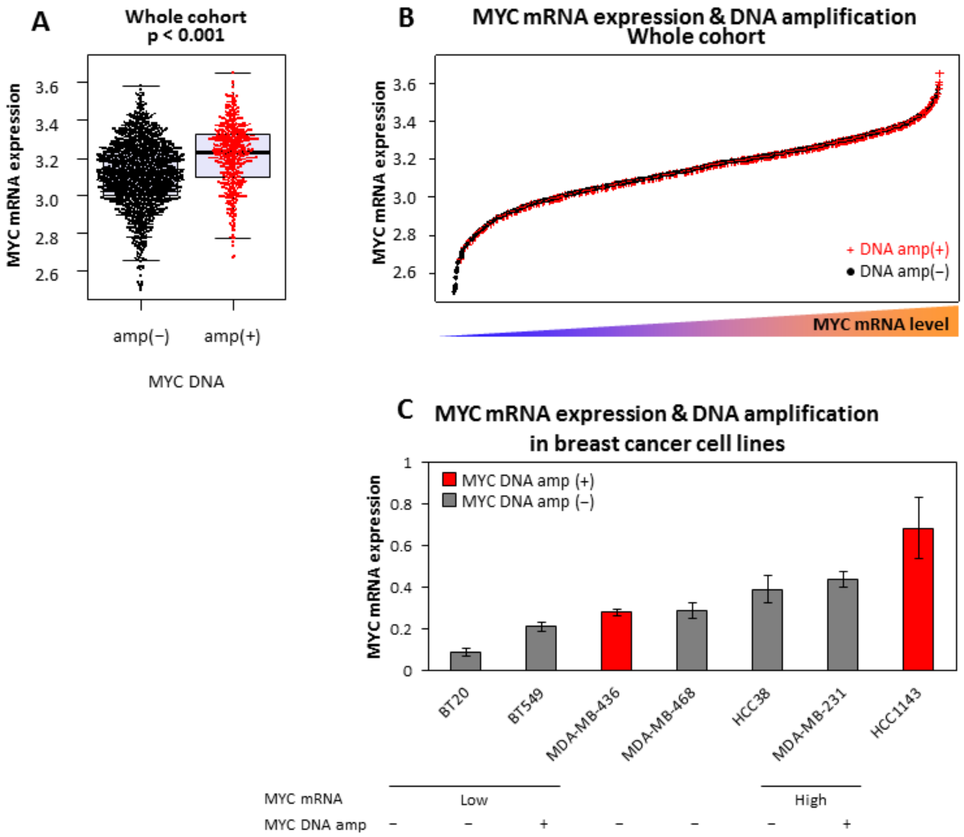


**Figure S1.** MYC mRNA expression and DNA amplification in the METABRIC whole breast cancer cohort and breast cancer cell lines: (**A**) MYC mRNA expression levels of MYC DNA non-amplified (amp(−)) and amplified (amp(+)) tumors, (**B**) MYC mRNA expression levels of MYC DNA non-amplified (DNA amp(−) in black) and amplified (DNA amp(+) in red) tumors. (**C**) MYC mRNA expression of MYC DNA non-amplified (DNA amp(−) in black column) and amplified (DNA amp(+) in red column) breast cancer cell lines.

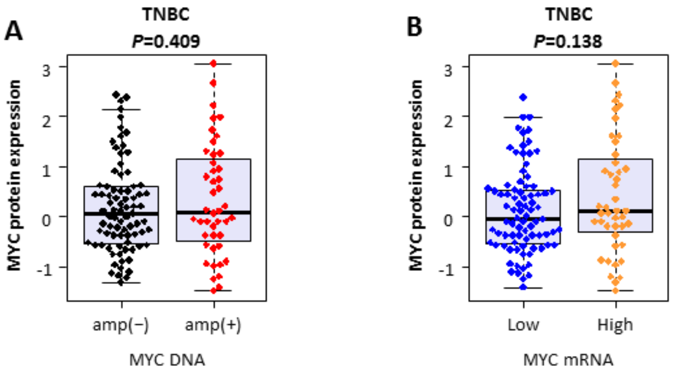


**Figure S2.** MYC protein expression by MYC amplification or mRNA expression in TCGA TNBC cohort: (**A**) MYC protein expression levels of MYC DNA non-amplified (amp(−)) and amplified (amp(+)) TNBCs, (**B**) MYC protein expression levels of MYC mRNA low and high TNBCs.

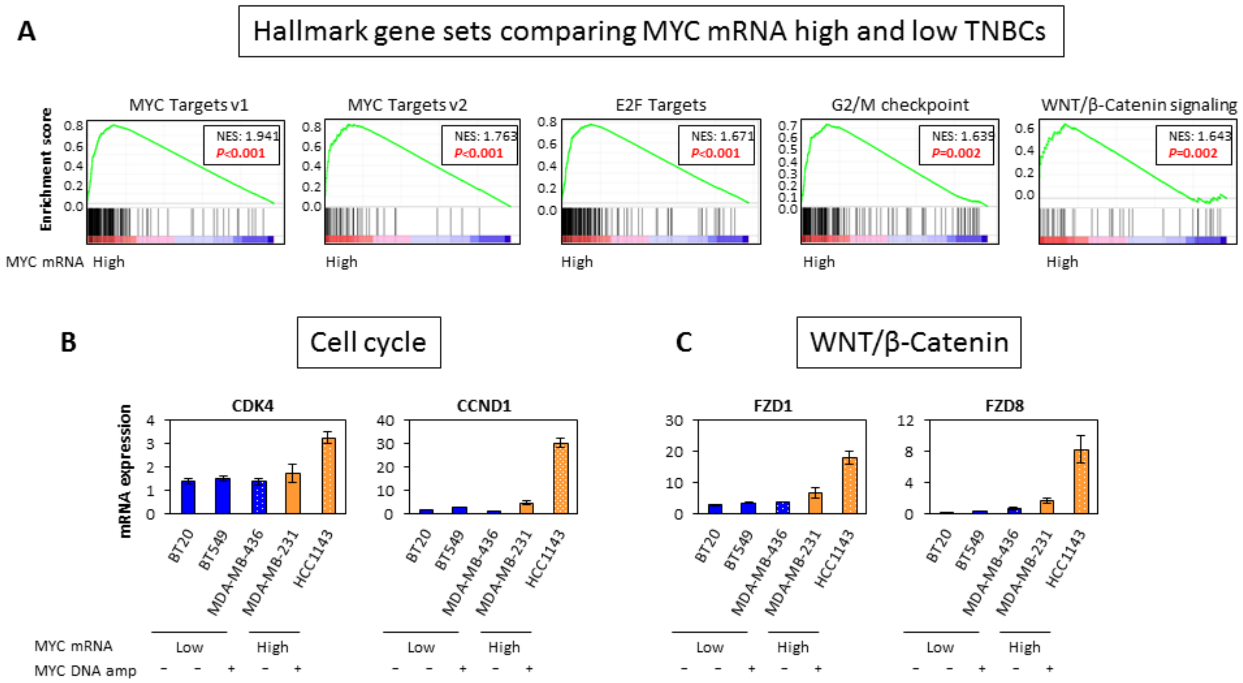


**Figure S3.** MYC targets, cell cycle, and WNT/β-Catenin genes by MYC mRNA expression in TNBC. (**A**) GSEA comparing MYC mRNA high and low expressing TNBCs in METABRIC cohort. (**B**) Cell cycle related genes expressions and (**C**) WNT/β-Catenin pathway genes expressions in MYC mRNA low (blue column) and high (orange column) TNBC cell lines.
